# Cost-Effectiveness of an Intensive Upper Limb Rehabilitation Therapy for Children With Unilateral Cerebral Palsy: An Economic Evaluation of a Randomized Controlled Trial

**DOI:** 10.36469/001c.94460

**Published:** 2024-04-11

**Authors:** Michael C. David, Hideki Higashi

**Affiliations:** 1 School of Medicine and Dentistry, Griffith University, Gold Coast, Australia; 2 The Daffodil Centre, The University of Sydney, a joint venture with Cancer Council NSW, Sydney, New South Wales, Australia; 3 School of Public Health, The University of Queensland, Herston , Australia

**Keywords:** cerebral palsy, cost-effectiveness analysis, randomized controlled trial, incremental cost-effective ratio, upper limb rehabilitation

## Abstract

**Background:** Unilateral cerebral palsy is a major cause of childhood disability and a substantial economic burden. Intensive group-based therapy, consisting of hybrid constraint-induced movement and bimanual therapies, has been shown to be effective in improving specific quality-of-life domains in children with this disability. Our objective in this study was to assess if this intervention was cost-effective compared with standard care.

**Methods:** An open-label, parallel, randomized controlled trial with an embedded economic evaluation of the intervention was conducted. A total of 47 children were randomized to either the intervention group (n = 27) or the standard care (n = 20) group. The effectiveness of the intervention was assessed using the Cerebral Palsy Quality of Life (Child) questionnaire across several domains. Nonparametric bootstrapping was used to quantify uncertainty intervals (UIs) for incremental cost-effectiveness ratios.

**Results:** The incremental cost-effectiveness ratios for the intervention were 273(95107 to 945)forPainandImpactofDisability,1071 (95% UI: -5718to4606) for Family Health and 1732(956448 to 8775)forAccesstoServices.Forthe4remainingdomains,theinterventionwasdominatedbystandardcare.Atawillingness−to−paythresholdof1000, only for the Pain and Impact of Disability domain was the intervention likely to have a probability of being cost-effective exceeding 0.75.

**Conclusions:** Other than the Pain and Impact of Disability domain, there was insufficient evidence demonstrating the intervention to be cost-effective over a 13-week time horizon.

## BACKGROUND

Cerebral palsy (CP) is a neurological condition caused by a lesion of the immature brain, leading to movement and posture disorders.^1^ It is the leading cause of childhood disability, with an estimated worldwide incidence rate ranging from 1.4 to 3.0 per 1000 live births.^2–4^ Unilateral cerebral palsy (UCP) is the most common type of CP in preterm children, with an incidence of 1 in 1300 live births.^5,6^ Among children with UCP, there are impairments in muscle tone, strength, sensation, and coordination of the impaired extremity that compromise unimanual and bimanual functionality. As a result, children with UCP have functional difficulties with grasping, reaching, releasing, and manipulating objects with the impaired upper limb.^7,8^ All contribute to reduced self-care, school, and household activities.^9^ Therapy to address and improve upper-limb activity is paramount in enhancing the quality of life (QoL) among children with UCP, as it relates to a person’s perception of feelings of well-being across several domains, such as physical, social, and emotional.^10^

In recent years, a range of targeted upper-limb therapy approaches has been developed for children with UCP. In their meta-analysis of nonsurgical upper-limb therapies for children with UCP, Sakzewski et al reported moderate to strong evidence supporting intensive models of modified constraint-induced movement therapy (CIMT), bimanual therapy, or hybrid-CIMT combining both approaches to improve upper-limb motor outcomes.^9^ These intensive models contrast with traditionally delivered individualized and distributed standard occupational therapy (SC). While little evidence supports this traditional approach, goal-directed home programs may offer the opportunity to increase the therapy dose, leading to improved upper-limb motor outcomes.^11–13^

Due to their cost, the healthcare system is greatly impacted by therapy interventions for UCP. It has been estimated that the medical costs for children with CP are 10 times the lifetime costs for children without CP.^14^ As a result, there is a need to conduct economic evaluations of existing therapies compared with new intensive models (hybrid-CIMT) to achieve optimum benefit from the available resources. Economic evaluations should take a societal perspective so that all relevant costs and effects contribute to the evaluation, regardless of who pays the costs or receives the benefits.^15–17^ New or additional therapies should be effective and cost-effective.^18,19^

Consequently, this study aimed to investigate the economic impact of hybrid-CMIT compared with SC from a societal perspective within a randomized controlled trial. We hypothesized that children who received hybrid-CMIT would have higher QoL than those in SC and that the former would be cost-effective.

## METHODS

### Participants

Children with UCP were recruited across Queensland, Australia, from January 2012 to June 2013. Potential study participants were identified through a population-based research database of more than 1300 children with CP at the Queensland Cerebral Palsy and Rehabilitation Research Centre, the Queensland Paediatric Rehabilitation Service, and the Cerebral Palsy Register. The recruitment process targeted publicly funded services and private practitioners with the expectation that the sample would be representative of children with UCP.

Children were eligible to be included if they (1) had a confirmed diagnosis of UCP and (2) had reduced upper-limb function due to predominant spasticity rather than dystonia and (3) were aged 5 to 16 years and (4) had sufficient cooperation and cognitive understanding to participate in the therapy activities. Children were ineligible if they (1) had fixed contracture or severe muscle spasticity in the designated muscle groups or (2) had previously undergone upper-limb surgery or (3) had received intramuscular botulinum toxin A injections within 6 weeks before baseline assessments.

Full ethical approval for the study was obtained from the Medical Ethics Committee of the University of Queensland (2011000553), the Royal Children’s Hospital Brisbane (HREC/11/QRCH/37), and the Cerebral Palsy League Ethics Committee (CPL-2012-004). Trial registration was obtained with the Australian and New Zealand Clinical Trials Registry (ACTRN12609000912280). Before entering the trial, informed written consent was obtained from all parents or guardians and assent from children (if ≥12 years of age). The published study protocol reports full details of the study methods.^20^ No subgroup analyses were predefined in the trial protocol.

### Study Design

The current economic analysis was integrated within an open-label, parallel randomized controlled trial that contrasted the efficacy of an intensive group therapy for the upper limb, implemented using a day camp approach (hybrid-CIMT),^20^ with a comparable dose of personalized occupational therapy given in the community (SC). This economic assessment was conducted from a societal viewpoint over 13 weeks, aligning with the trial’s duration. It encompassed all expenses (whether direct or indirect) and outcomes of each therapy method.

Children were randomized within matched pairs to either hybrid-CIMT or standard occupational therapy care (SC). To maximize the homogeneity of the sample and minimize group differences at baseline, children were matched according to age (12-month bands), Gross Motor Function Classification Scale (GMFCS)^21^ and Manual Ability Classification System (MACS).^22^ The GMFCS classifies a child’s ability to perform self-initiated movements related to sitting and walking across 5 levels, while the MACS classifies a child’s ability to handle objects in their daily activities. The MACS is a simple, 5-level, ordinal grading system created to categorize the fine motor ability of children with CP in the 4- to 18-year age range, with high psychometric properties.^23^ The randomization was implemented by sealed envelopes using computer-generated random numbers balanced in blocks of 2. Group allocation was generated by the study statistician and unsealed by nonstudy personnel.

### Sample Size

An appropriate sample size determination for this study was based on having sufficient power to compare the functional effects of hybrid-CIMT and SC therapies at 13 weeks. Based on data from a previous study,^24^ a mean difference of 7 percentage points (SD = 9) on the Melbourne Assessment of Unilateral Upper Limb Function was postulated as the minimum difference likely to have clinical implications. With an α of .05 and a power of .80, it was calculated that a sample size of 25 subjects in each group (n = 50) was required.

### Intervention and Standard Care Therapies

Participants received either a hybrid-CIMT or an SC rehabilitation approach. While both groups received a dosage of 45 hours of upper-limb training, they differed by (1) delivery method (group vs individualized), (2) intensity (high intensity over 2 weeks vs low intensity over 12 weeks), and (3) environment (community-based camp vs community center/rehabilitation unit or home).

For the hybrid-CIMT group, participation was by attendance at 1 of 2 camps. The first camp was held in January 2012 and the second in mid-2012. Therapy consisted of intensive direct upper-limb training spread over 2 weeks using a novel circus theme to enhance children’s motivation for engagement and participation. Children in this group attended a community facility, Flipside Circus in Brisbane, Australia, for 6 hours a day, coordinated by a team of occupational therapists and physiotherapists. In the first week, intense tasks were given to the children to utilize their impaired arm through the constraint of their unimpaired arm with an individually made glove. The glove was constructed from breathable fabric with a volar plastic insert to prevent grasp and was removed for no more than 15 minutes per day. In the second week, the glove was removed, and adopting the strategy used by Gordon et al^25^ encouraged using both hands to implement bimanual tasks. All tasks were analyzed and selected to target specific movement components required for goal achievement and were novel, fun, and motivating, and fostered self-generating voluntary repetition of bimanual tasks.

Children in the SC group received individualized occupational therapy (focused on improving upper-limb function) of equal dosage to those in the hybrid-CIMT group. This dosage consisted of 6 weekly sessions of 1.5 hours of individual direct therapy in a hospital or community, and other treatments in their homes. As part of the latter component, families were provided with a 36-hour home program to practice goal areas from the commencement of the individual therapy sessions (30 minutes daily for 6 days/week for 12 weeks).

### Cost Measures

The economic evaluation aimed to determine and compare the costs associated with the 2 groups and relate these costs to effects. First, the following relevant categories of resource utilization were identified: (1) direct intervention costs, (2) direct non-intervention costs, and (3) indirect costs. Second, the volume of each category for each child was measured, and resource costs measured these volumes. Direct intervention costs consisted of (1) Flipside Circus registration fees and (2) healthcare staff salaries, the latter particular to the hybrid-CIMT group. Direct nonintervention costs consisted of (1) accommodation and travel costs and (2) Flipside Circus consumables (eg, restraint gloves), with the latter relating to the reimbursement of costs incurred by families of children participating in the trial. Indirect costs, consisting of (1) Flipside Circus catering and (2) productivity losses, were linked to the days primary caregivers took off work. Information on the days primary caregivers missed work was gathered during the trial through phone interviews. These missed days were then translated into productivity costs using the median earnings data from Australia, categorized by age, gender, and highest educational attainment.^26^ An estimate of the monthly productivity cost per participant was derived based on the participants’ last contact date. While all SC costs were individualized, hybrid-CIMT costs consisted of individual and aggregate data. All costs were valued in 2020 Australian dollars (AUD).

### Effectiveness Measures

The primary caregiver completed the parent proxy of the Cerebral Palsy QoL Child Questionnaire (CPQoL-Child) to assess the child’s QoL from the parents’ perspectives. The questionnaire contains 66 items across 7 domains:

1. Access to Services
2. Emotional Well-Being
3. Family Health
4. Feelings about Functioning
5. Participation and Physical Health
6. Pain and Impact of Disability
7. Social Well-Being and Acceptance

*Access to Services* pertains to the availability and ease of use of community resources or facilities that assist parents and children with CP. *Emotional Well-Being* encompasses feelings of happiness, contentment with accomplishments, and maintaining a positive emotional condition. *Family Health* includes parental happiness and parents’ and primary caregivers’ physical and emotional well-being. *Feelings about Functioning* refers to communicating with others in the family and community and performing daily activities such as feeding, dressing, and toileting. *Participation and Physical Health* refers to involvement in school, sports and community activities; adequate motor skills; the capacity to use aids/equipment; and bodily health and wellness. *Pain and Impact of Disability* refers to physical pain or discomfort and pain related to therapy. *Social Well-Being and Acceptance* refers to interactions with peers, family, and community members. Social participation within this domain emphasizes social connections and relationships, the ability to engage in social endeavors, and the experience of social acceptance.^24^

Each question on this form is worded as, “How do you think your child feels about…” and requires an answer from 1 to 9 where 1 = Very unhappy, 3 = Unhappy, 5 = Neither happy nor unhappy, 7 = Happy, and 9 = Very happy. The scoring method consisted of 2 stages. Initially, each item’s raw scores were converted to a scale ranging from 0 to 100 points. Subsequently, the scores for each domain were determined by taking the average of the item scores. In all domains except for Pain and Impact of Disability, a higher score signifies a superior QoL. The CPQoL-Child boasts outstanding psychometric qualities^27^ and a test-retest reliability between .76 and .89 for its 7 domains.^28^ Since QoL is described as “holistic well-being,” scores are presented by domain rather than a collective sum.^29^

### Statistical Analysis

The analyses reported here were all planned *a priori.* There were no interim analyses or stopping rules. The primary analysis was intention to treat, with children analyzed in their randomized group irrespective of whether they completed the trial. We used multiple imputation with chained equations approach to compensate for missing data to create 10 imputed data sets and combined these results with Rubin’s rules.^30^ As multiple imputation has consistently been shown to be a valid approach in handling missing data, only estimates obtained by multiple imputation are reported.^31^ All imputed data were assumed missing at random. Baseline characteristics of participants are reported as counts (percentages). Due to non-normality, therapy costs per child are reported as medians (interquartile ranges [IQRs]) and compared using the Mann-Whitney *U* test. Independent Student *t*-tests with unequal variance were used to compare change (baseline to follow-up) in mean QoL domain scores between groups. As all comparisons were of interest, there was no adjustment for multiple testing.^32^

To assess hybrid-CIMIT cost-effectiveness, incremental cost-effectiveness ratios (ICERs) were calculated. ICERs were calculated for each CPQoL-Child domain by dividing the incremental cost (the difference in mean cost between hybrid-CIMT and SC) by the incremental effect (the difference in mean QoL score between hybrid-CIMT and SC). From a societal viewpoint, the ICER factored in direct and indirect costs, encompassing those within and outside the healthcare sector. Except for the pain and impact of disability domain, ICERs for each domain were understood as the additional cost per extra point on the domain score.^33^ Conversely, for pain and impact of disability, a lower QoL score indicates less pain. Non-parametric bootstrapping with 10,000 iterations was used to quantify uncertainty in estimating differences between COMBiT and SC groups for mean costs, mean effects, and ICERs by 95% UIs. We generated cost-effectiveness acceptability curves to evaluate the probability that hybrid-CIMIT is cost-effective across different willingness-to-pay limits.

All tests for significance were 2-sided, with a *P* value below .05 denoting statistical significance. The analyses were carried out using STATA version 18.0.

## RESULTS

### Participants

**Figure 1.**
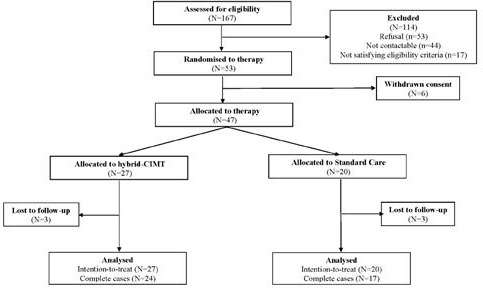
Participant Flowchart Abbreviation: hybrid-CIMT, group-based therapy combining modified constraint-induced movement therapy and bimanual therapy.

One hundred sixty-seven children were assessed for eligibility, and 114 (68%) were excluded, either due to refusal (n = 53; 2%), being noncontactable (n = 44; 26%), or not satisfying eligibility criteria (n = 17; 10%), as shown in **Figure 1**. Fifty-three children aged between 4.7 and 12.5 years were randomized to either the hybrid-CIMT group (n = 28) or the SC group (n = 25). Our final sample consisted of 47 children, 27 in the hybrid-CIMT group and 20 in the SC group, due to the caregivers of 6 children withdrawing consent after allocation but before receiving therapy.

Sociodemographic characteristics of the children allocated to each group are reported in **Table 1**. Complete data were obtained for the characteristics of age, Gross Motor Function Classification Scale, hemiplegia (left- or right-sided), Manual Ability Classification System, sex, and Statistics Socio-Economic Indexes for Areas (SEIFA), and UCP, while family income had 3 cases of missing data. Overall, participating children were more likely to be male (70%), have parents with a combined annual family income of less than $50 000 (80%), and come from low to middle SEIFA locations (81%). The sample prevalence of right-sided and left-sided UCP was comparable. No major differences in baseline characteristics between the 2 therapy groups were seen to exist.

**Table 1.** Baseline Characteristics of Children and Their Families by Therapy Group

**Characteristic**	**Total**	**Hybrid-CIMT**	**Standard Care**
	**(N = 47), n (%)**	**(N = 27), n (%)**	**(N = 20), n (%)**
Age (years)			
4-8	28 (60)	15 (57)	13 (65)
9-13	19 (40)	12 (44)	7 (35)
Family income^a^			
<$25 000	22 (50)	11 (44)	11 (58)
25 000−49 999	13 (30)	8 (32)	5 (26)
50 000-$74 999	8 (18)	5 (20)	3 (16)
≥$75 000	1 (2)	1 (4)	0 (0)
SEIFA^b^			
Low	19 (40)	10 (37)	9 (45)
Medium	19 (40)	11 (41)	8 (40)
High	9 (19)	6 (22)	3 (15)
Sex			
Female	14 (30)	8 (30)	6 30)
Male	33 (70)	19 (70)	14 (70)
Unilateral cerebral palsy			
Left-sided	22 (47)	12 (44)	10 (50)
Right-sided	25 (53)	15 (56)	10 (50)

^a^Three values are missing.

^b^Socioeconomic index for area.

Abbreviations: hybrid-CIMT, group-based therapy combining modified constraint-induced movement therapy and bimanual therapy; SEIFA, Statistics Socio-Economic Indexes for Areas.

### Costs

**Table 2** presents median (IQR per-child costs incurred in administering hybrid-CIMT and SC therapies. The median cost per child was $5733 (IQR: $2344-$7494) for those in the hybrid-CIMT group, while the median cost per child was $2970 (IQR: $2970-$3855) for those in the SC group. This difference was found to be statistically significant (*P* = .016). Of the 3 cost types, indirect costs were the most comparable across therapy groups, with a median cost per child of $2964 (IQR: $272-$2964) and $2288 ($1615-$2961) for hybrid-CIMT and SC, respectively. While this comparison between therapies was found to be nonsignificant ($2964 vs $2288; *P* = .117), this was not the case for direct intervention costs ($2056 vs $690; *P* < .001) or direct non-intervention costs ($713 vs $0; *P* = .002). Except for direct intervention costs, there was a relatively large amount of uncertainty in these results, as indicated by the wide IQRs for accommodation/travel and productivity cost.

**Table 2.** Comparison of Costs Between Hybrid-CIMT and Standard Care Groups Costs

	**Therapy Costs (AUD)**				
	**Hybrid-CIMT (n = 27)**		**SC (n = 20)**		***P* Value^b^**
	**Median**	**IQR**	**Median**	**IQR**	
Direct medical costs					
Flipside Camp registration	425	425-425	0	0-0	NA
Healthcare staff salaries	1631	1631-1631	690	392-761	<.001
Subtotal	2056	2056-2056	690	392-761	<.001
Direct non-medical costs					
Accommodation and travel	697	0-2458	0	0-534	.078
Flipside Camp consumables	16	16-16	0	0-0	NA
Subtotal	713	16-2474	0	0-534	.002
Indirect costs					
Flipside Camp catering	272	272-272	0	0-0	NA
Productivity cost	2692	0-2692	2288	1615-2961	.025
Subtotal	2964	272-2964	2288	1615-2961	.117
Total cost	5733	2344-7494	2970	2376– 3855	.016

^a^Mann-Whitney *U* for difference in change scores between therapy groups.

Abbreviations: AUD, Australian dollars (2020); hybrid-CIMT, group-based therapy combining modified constraint-induced movement therapy and bimanual therapy; IQR, interquartile range; NA, not applicable; SC, standard care therapy.

### Effectiveness

With the inclusion of imputed data, scores for the 7 CPQoL-Child primary domains are presented in **Table 3** for both groups at baseline and at 13-week follow-up. Without exception, higher QoL scores were seen across all domains within the SC group. Besides emotional well-being, QoL scores improved across all domains within the hybrid-CIMT group, with the largest improvement being for participation and physical health (follow-up: mean [SD] = 70.94 [11.59] vs baseline: mean [SD] = 66.33 [14.57]). Similarly, the largest improvement within the SC group was also seen in the same domain (follow-up: 79.04 [11.59] vs baseline: 71.48 [15.49]). The difference-in-change estimate for pain and impact of disability was the only intervention effect that was statistically significant (*P* = .024), with scores improving by 0.31 (SD = 16.09) units in the hybrid-CIMT group and worsening by 8.81 (SD = 10.63) units in the SC group.

**Table 3.** Difference-in-Difference Analyses Between Hybrid-CIMT and Standard Care Groups for Intervention Effect During 13-Week Follow-up

**CPQoL-⁠Child Domain**	**Therapy**						**Difference- in-⁠Difference *P* Value^c^**
	**Hybrid-CIMT(n=27)**			**SC (n=20)**			
	**Baseline Mean (SD)**	**Follow-up Mean (SD)**	**Change Mean^a^ (SD)**	**Baseline Mean (SD)**	**Follow-up Mean (SD)**	**Mean Change^b^ (SD)**	
1. Functioning	68.71 (10.03)	70.49 (12.51)	1.78 (9.45)	74.95 (14.09)	78.29 (11.12)	3.34 (14.74)	.681
2. Participation and Physical Health	66.33 (14.57)	70.94 (11.59)	4.61 (13.64)	71.48 (15.49)	79.04 (10.13)	7.56 (14.32)	.481
3. Access to Services	63.40 (18.37)	65.06 (14.45)	1.66 (14.48)	64.70 (15.01)	64.95 (18.73)	0.25 (19.58)	.789
4. Emotional Well- Being	79.63 (11.18)	79.52 (11.21)	-0.11 (10.71)	83.44 (15.09)	85.96 (9.23)	2.52 (14.72)	.503
5. Impact of Disability and Family Health	65.97 (17.54)	66.28 (16.74)	0.31 (11.45)	76.41 (15.56)	74.36 (10.54)	-2.05 (12.54)	.513
6. Pain^d^	27.72 (16.05)	27.41 (17.45)	-0.31 (16.09)	27.05 (16.34)	35.86 (12.56)	8.81 (10.63)	.024
7. Social Well-Being and Acceptance	79.16 (11.43)	81.10 (10.73)	1.94 (12.20)	83.42 (11.01)	86.99 (8.70)	3.56 (11.51)	.643

^a^The mean change in quality-of-life scores under hybrid-CIMT therapy between baseline and follow-up.

^b^The mean change in quality-of-life scores under standard care therapy between baseline and follow-up.

^c^Paired *t*-test for difference in change scores between therapy groups.

^d^Unlike other CPQoL-Child Domains, higher scores on Pain indicates a reduction in quality of life, while lower scores indicate an improvement in quality of life.

Abbreviations: CPQoL-Child, Cerebral Palsy Quality of Life Child Questionnaire; CI, confidence interval; hybrid-CIMT, combined bimanual training and modified constraint-induced movement therapies; SC, standard care group.

### Cost-Effectiveness

Point and interval estimates of domain ICERs are reported in **Table 4**. For all domains, the mean incremental cost was $2527 per child. For 4 domains, Emotional Well-Being, Functioning, Participation and Physical Health and Social Well-Being and Acceptance, hybrid-CIMT was dominated by SC, with hybrid-CIMT being less effective and more costly than SC. A base-case analysis of the 3 remaining domains indicated hybrid-CIMT to be more cost-effective in reducing pain and impact of disability than improvements relating to Family Health or Access to Services. For the domain of Pain and Impact of Disability, the cost to reduce pain and impact of disability by 1 QoL unit was estimated to be $273 (95% UI: $107-$945).

**Table 4.** Incremental Cost-Effectiveness for an Additional Unit of QoL Gained During 13-Week Follow-up

**CPQoL-Child Domain**	**Incremental Cost per Child (AUD)**		**Incremental Effect per Child**		**ICER (AUD)**
	**Mean**	**UI**	**Mean**	**UI**	**Mean (UI)^a^**
1. Access to Services	2528	1373-3638	1.46	-8.43 to 11.90	1732 (-⁠6448 to 8775)
2. Emotional Well-Being	2527	1325-3729	-2.63	-10.18 to 4.25	Dominated
3. Functioning	2527	1325-3729	-1.56	-8.91 to 5.30	Dominated
4. Family Health	2527	1325-3729	2.36	-1.99 to 11.20	1071 (-5718 to 4606)
5. Pain and Impact of Disability	2527	1325-3729	-9.24^b^	-16.72 to -2.14	273 (107 \to 945)^c^
6. Participation and Physical Health	2527	1325-3729	-2.95	-10.89 to 4.75	Dominated
7. Social Well-⁠Being and Acceptance	2527	1325-3729	-1.62	-8.12 to 4.88	Dominated

^a^Uncertainty interval obtained from 10 000 nonparametric bootstrapping iterations.

^b^Unlike other CPQoL–Child Domains, higher scores on Pain indicates a reduction in QoL.

^c^ICER estimate indicates the cost of averting or reducing Pain score by 1 unit on a 0-100 QoL scale.

Abbreviations: AUD, Australian dollars; ICER, incremental cost-effectiveness ratio; QoL, quality of life; UI, 95% uncertainty interval.

**Figure 2.**
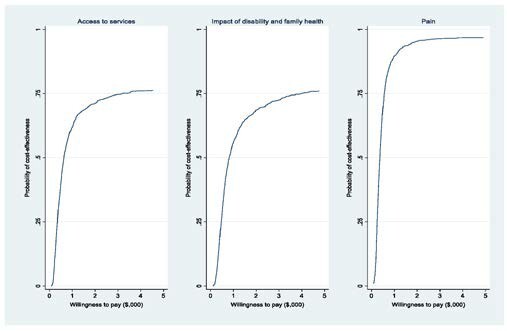
Cost-Effectiveness Acceptability Cures for the Domains of Access to Services, Impact of Disability and Family Health, and Pain

In cases where the hybrid-CIMT intervention resulted in higher costs and QoL scores than SC, cost-effectiveness can be assessed based on a decision maker’s willingness to pay (WTP) for a beneficial change of 1 QoL unit. The intervention can be considered cost-effective if this cost falls below the WTP value. For the 3 domains where SC did not outperform hybrid-CIMT, the outcomes from a probabilistic sensitivity analysis, visualized as a cost-effectiveness acceptability curve, are depicted in **Figure 2**. This curve showcases the program’s probability of cost-effectiveness against different WTP levels. For pain and impact of disability, there was a .82 probability of hybrid-CIMT being cost-effective at a WTP of $1000. However, for the domains of access to services and family health, the probabilities were both below .50.

For each of the 3 domains in which SC did not dominate hybrid-CIMT, the results of a probabilistic sensitivity analysis in the form of cost-effectiveness acceptability curve, whereby the likelihood of the program being cost-effective is plotted against varying levels of WTP, are shown in **Figure 2**. For Pain and Impact of Disability, the probability of hybrid-CIMT being cost-effective at a WTP of $1000 was .82, while both access to services and family health were less than .50.

## DISCUSSION

This study reports an economic evaluation of hybrid-CIMT compared with SC in improving the QoL in children with congenital UCP. Besides the domain of Emotional Well-Being, QoL scores improved under hybrid-CIMT therapy. However, hybrid-CIMT was substantially more costly than SC in the base-case analysis, driven by staffing salaries. Thus, compared with SC, hybrid-CIMT was deemed cost-effective solely for Access to Services, Family Health, and Pain and Impact of Disability. For the remaining domains, SC surpassed hybrid-CIMT. This aligns with results found in other studies that contrasted hybrid-CIMT with SC.^34–37^

Our study has several strengths, in particular, its originality. To our knowledge, it is one of the first economic evaluations to compare an intensive group-based model of therapy embedded in a community leisure facility (hybrid-CIMT) with individualized SC in children with UCP from a societal perspective. An important strength of the current study was that it was conducted alongside a randomized controlled trial of the 2 therapies. It compared equal treatment dosages to minimize subject differences in a matched pairs design. Thus, the data are less prone to sources of bias and confounding than those generated by nonrandomized study design.^38,39^ A further strength is that this was a pragmatic study, reflecting a situation close to clinical practice.^40^

This study also presents some caveats. First, no algorithm was available for aggregating domain QoL scores into a single index, as per the SF-36 or the EuroQol questionnaire (EQ-5D).^41^ Drawing from the WHO’s definition of QoL as “wide-ranging areas of well-being,” the CPQoL-Child questionnaire was crafted to be specific to the condition. As a result, combining domain QoL scores was deemed unsuitable,^42^ leading our cost-effectiveness analysis of hybrid-CIMT therapy to focus on specific domains. Additionally, the potential presence of missing QoL data at the 13-week checkpoint might challenge the validity of our findings.^43^ However, inferences drawn from the nonreported complete-case analysis did not differ from inferences drawn from our analysis using multiple imputation, suggesting the results are robust to the influence of missing data.^44,45^ Third, while both groups appeared to be balanced concerning participant characteristics, this was not the case with mean QoL domain scores at baseline, each indicating higher QoL among those in the SC group, irrespective of the domain. Consequently, one would expect an increased likelihood of QoL scores emanating from the hybrid-CIMT group to regress to the mean. As a significant effect was detected only for the domain of Pain and Impact of Disability, it is unlikely that this “statistical phenomenon” substantially biased the intervention effect.^46^ Fourth, the estimation of caregiver productivity costs centerd solely on primary caregiver information, neglecting secondary caregivers or other family members who might have also taken time off for caregiving duties. The valuation of lost caregiver workdays relied on national survey figures since specific earnings data were not gathered during the trial. Such constraints could lead to an underrepresentation of the actual costs of patients in both groups. While self-reporting is often viewed as the benchmark for QoL assessment,^47^ hybrid-CIMT might offer a more extensive array of advantages than what a single QoL domain measure can capture. This includes nonhealth benefits like the experience and satisfaction of both patients and families with the care received and even the contentment of the provider.^48^ Such potential benefits of the hybrid-CIMT approach were not highlighted in this research.

The lack of notable benefit in terms of QoL from hybrid-CIMT drives the main conclusion of this analysis. Only for the domain of Pain and Impact of Disability did hybrid-CIMT appear substantially more cost-effective than SC. As such, this economic evaluation does not support using hybrid-CIMT instead of SC as an appropriate use of resources. Intensive group-based models of therapy hybrid-CIMT may be a cost-effective therapy option under varying circumstances that differ from this study, one possibility being a longer follow-up period.

Our results are limited to a certain extent by our inability to interpret cost-effectiveness associated with changes on a generic QoL scale. While we agree that condition-specific QoL measures are developed across broad domains of well-being based on the WHO definition of QoL, cost-utility outcomes may need to be considered in future economic analyses. As participants were required to be between 5 and 16 years of age, generalizability to children 4 years or younger was not possible. Also, it is recommended that future research incorporates WTP measures in their analyses to ascertain what stakeholders are prepared to fund. This is highly relevant given current international funding priorities for disability schemes such as Australia’s National Disability Insurance Scheme.^41,49^ Ongoing research should examine the long-term effectiveness of hybrid-CIMT and strategies to improve the cost-effectiveness of hybrid-CIMT. In addition, and as recommended by Gupta et al, the use of decision trees and Markov models should be encouraged as they make use of evidence from several sources, thereby increasing generalizability when compared with evidence from a single trial.^50^

## CONCLUSIONS

This economic evaluation found that an intensive group model delivered in a community leisure facility such as hybrid-CIMT compared with an individualized distributed model such as SC is not cost-effective for children with UCP. The results of this study should be used to improve the quality of care for children with this UCP. Nevertheless, it also needs to be realized that decisions relating to health service delivery are not based solely on cost-effectiveness analysis, and any final recommendation for or against intensive group-delivered models will also be influenced by issues such as acceptance, equity, and feasibility.^16,33^

### Availability of Data and Materials

The data sets used and/or analyzed during the present study are available from the corresponding author upon reasonable request.

### Code Availability

The code used for the present study is available from the corresponding author upon reasonable request.

### Conflict of Interest

The authors declare that they have no conflict of interest.

### Ethics Approval

Ethics approval to conduct the study and experimental protocols was approved by the Medical Ethics Committee of The University of Queensland (2011000553), The Royal Children’s Hospital Brisbane (HREC/11/QRCH/37) and The Cerebral Palsy League Ethics Committee (CPL-2012-004). Consequently, all methods utilized in the study were carried out in accordance with relevant guidelines and regulations. The participants in the study were provided written and verbal information about the study. They were also informed that their participation was voluntary. Written informed consent was obtained from the parents and guardians of each study participant. All the requirements of the Helsinki Declaration were fulfilled.

## References

[ref-298463] Rosenbaum P., Paneth N., Leviton A.. (2007). A report: the definition and classification of cerebral palsy April 2006. Dev Med Child Neurol.

[ref-298464] Sadowska Małgorzata, Sarecka-Hujar Beata, Kopyta Ilona (2020). Evaluation of risk factors for epilepsy in pediatric patients with cerebral palsy. Brain Sciences.

[ref-298465] Longo Egmar, Regalado Isabelly Cristina, Galvão Elida Rayane, Ferreira Haryelle Nárima, Badia Marta, Baz Begonã Orgaz (2020). I want to play: children with cerebral palsy talk about their experiences on barriers and facilitators to participation in leisure activities. Pediatric Physical Therapy.

[ref-298466] Patel Dilip R., Neelakantan Mekala, Pandher Karan, Merrick Joav (2020). Cerebral palsy in children: a clinical overview. Translational Pediatrics.

[ref-298467] Hagberg B, Hagberg G, Beckung E, Uvebrant P (2001). Changing panorama of cerebral palsy in Sweden: prevalence and origin in the birth year period 1991–94. Acta Paediatrica.

[ref-298468] Stanley F., Blair E., Alberman E. (2000). Clinics in Developmental Medicine.

[ref-298469] Hoare Brian J, Wallen Margaret A, Thorley Megan N, Jackman Michelle L, Carey Leeanne M, Imms Christine (2019). Constraint-induced movement therapy in children with unilateral cerebral palsy. Cochrane Database Syst Rev.

[ref-298470] Sakzewski LEANNE, Carlon STACEY, Shields NORA, Ziviani JENNY, Ware ROBERT S, Boyd ROSLYN N (2012). Impact of intensive upper limb rehabilitation on quality of life: a randomized trial in children with unilateral cerebral palsy. Developmental Medicine & Child Neurology.

[ref-298471] Sakzewski Leanne, Ziviani Jenny, Boyd Roslyn (2009). Systematic review and meta-analysis of therapeutic management of upper-limb dysfunction in children with congenital hemiplegia. Pediatrics.

[ref-298472] Bjornson K. F., McLaughlin J. F. (2001). The measurement of health-related quality of life (HRQL) in children with cerebral palsy. European Journal of Neurology.

[ref-298473] Novak Iona, Morgan Cathy, Adde Lars, Blackman James, Boyd Roslyn N., Brunstrom-Hernandez Janice, Cioni Giovanni, Damiano Diane, Darrah Johanna, Eliasson Ann-Christin, de Vries Linda S., Einspieler Christa, Fahey Michael, Fehlings Darcy, Ferriero Donna M., Fetters Linda, Fiori Simona, Forssberg Hans, Gordon Andrew M., Greaves Susan, Guzzetta Andrea, Hadders-Algra Mijna, Harbourne Regina, Kakooza-Mwesige Angelina, Karlsson Petra, Krumlinde-Sundholm Lena, Latal Beatrice, Loughran-Fowlds Alison, Maitre Nathalie, McIntyre Sarah, Noritz Garey, Pennington Lindsay, Romeo Domenico M., Shepherd Roberta, Spittle Alicia J., Thornton Marelle, Valentine Jane, Walker Karen, White Robert, Badawi Nadia (2017). Early, accurate diagnosis and early intervention in cerebral palsy. JAMA Pediatrics.

[ref-298474] Hung Ya-Ching, Spingarn Aryeh, Friel Kathleen M., Gordon Andrew M. (2020). Intensive unimanual training leads to better reaching and head control than bimanual training in children with unilateral cerebral palsy. Physical & Occupational Therapy In Pediatrics.

[ref-298475] Wallen MARGARET, Ziviani JENNY, Naylor OLIVIA, Evans RUTH, Novak IONA, Herbert ROBERT D (2011). Modified constraint-induced therapy for children with hemiplegic cerebral palsy: a randomized trial. Developmental Medicine & Child Neurology.

[ref-298476] Centers for Disease and Prevention Data and statistics for Cerebral Palsy.

[ref-298477] Byford S., Raftery J. (1998). Economics notes: perspectives in economic evaluation. BMJ.

[ref-298478] Neumann Peter J., Sanders Gillian D., Russell Louise B., Siegel Joanna E., Ganiats Theodore G. (2016). Cost-effectiveness in Health and Medicine.

[ref-298479] Jönsson Bengt (2009). Ten arguments for a societal perspective in the economic evaluation of medical innovations. The European Journal of Health Economics.

[ref-298480] Gaziano Thomas A, Galea Gauden, Reddy K Srinath (2007). Scaling up interventions for chronic disease prevention: the evidence. The Lancet.

[ref-298481] Sakzewski Leanne, Miller Laura, Ziviani Jenny, Abbott David F, Rose Stephen, Macdonell Richard A L, Boyd Roslyn N (2015). Randomized comparison trial of density and context of upper limb intensive group versus individualized occupational therapy for children with unilateral cerebral palsy. Developmental Medicine & Child Neurology.

[ref-298482] Boyd Roslyn N, Ziviani Jenny, Sakzewski Leanne, Miller Laura, Bowden Joanne, Cunnington Ross, Ware Robert, Guzzetta Andrea, AL Macdonell Richard, Jackson Graeme D, Abbott David F, Rose Stephen (2013). COMBiT protocol of a randomized comparison trial of combined modified constraint-induced movement therapy and bimanual intensive training with a distributed model of standard upper limb rehabilitation in children with congenital hemiplegia. BMC Neurology.

[ref-298483] Eliasson Ann-Christin, Krumlinde-Sundholm Lena, Rösblad Birgit, Beckung Eva, Arner Marianne, Öhrvall Ann-Marie, Rosenbaum Peter (2007). The Manual Ability Classification System (MACS) for children with cerebral palsy: scale development and evidence of validity and reliability. Developmental Medicine & Child Neurology.

[ref-298484] Palisano Robert, Rosenbaum Peter, Walter Stephen, Russell Dianne, Wood Ellen, Galuppi Barbara (1997). Development and reliability of a system to classify gross motor function in children with cerebral palsy. Developmental Medicine & Child Neurology.

[ref-298485] Piscitelli Daniele, Ferrarello Francesco, Ugolini Alessandro, Verola Sofia, Pellicciari Leonardo (2021). Measurement properties of the Gross Motor Function Classification System, Gross Motor Function Classification System-Expanded & Revised, Manual Ability Classification System, and Communication Function Classification System in cerebral palsy: a systematic review with meta-analysis. Developmental Medicine & Child Neurology.

[ref-298486] Boyd R. (2004). The Central and Peripheral Effects of Botulinum Toxin A in Children With Cerebral Palsy.

[ref-298487] Gordon Andrew M, Schneider Jennifer A, Chinnan Ashley, Charles Jeanne R (2007). Efficacy of a hand–arm bimanual intensive therapy (HABIT) in children with hemiplegic cerebral palsy: a randomized control trial. Developmental Medicine & Child Neurology.

[ref-298488] Commonwealth of Australia (2020). Employee Earnings, August 2020.

[ref-298489] Carlon Stacey, Shields Nora, Yong Katherine, Gilmore Rose, Sakzewski Leanne, Boyd Roslyn (2010). A systematic review of the psychometric properties of Quality of Life measures for school aged children with cerebral palsy. BMC Pediatrics.

[ref-298490] Waters Elizabeth, Davis Elise, Mackinnon Andrew, Boyd Roslyn, Graham H Kerr, Kai Lo Sing, Wolfe Rory, Stevenson Richard, Bjornson Kristie, Blair Eve, Hoare Peter, Ravens-Sieberer Ulrike, Reddihough Dinah (2007). Psychometric properties of the quality of life questionnaire for children with CP. Developmental Medicine & Child Neurology.

[ref-298491] Davis Elise, Waters Elizabeth, Mackinnon Andrew, Reddihough Dinah, Graham H Kerr, Mehmet-Radji Ozlem, Boyd Roslyn (2006). Paediatric quality of life instruments: a review of the impact of the conceptual framework on outcomes. Developmental Medicine & Child Neurology.

[ref-298492] Mainzer Rheanna, Apajee Jemishabye, Nguyen Cattram D., Carlin John B., Lee Katherine J. (2021). A comparison of multiple imputation strategies for handling missing data in multi-item scales: guidance for longitudinal studies. Statistics in Medicine.

[ref-298493] Rubin D.B. (2009). Multiple imputation for nonresponse in surveys.

[ref-298494] Greenland Sander, Hofman Albert (2019). Multiple comparisons controversies are about context and costs, not frequentism versus bayesianism. European Journal of Epidemiology.

[ref-298495] Drummond M.F., Sculpher M.J., Torrance G.W., O’Brien B.J., Stoddart G.L. (2015). Methods for the Economic Evaluation of Health Care Programmes.

[ref-298496] Eugster-Buesch Francisca, de Bruin Eling D., Boltshauser Eugen, Steinlin Maja, Küenzle Christoph, Müller Elisabeth, Capone Andrea, Pfann Renat, Meyer-Heim Andreas (2012). Forced-use therapy for children with cerebral palsy in the community setting: a single-blinded randomized controlled pilot trial. Journal of Pediatric Rehabilitation Medicine.

[ref-298497] Hoare Brian, Greaves Susan (2017). Unimanual versus bimanual therapy in children with unilateral cerebral palsy: same, same, but different. Journal of Pediatric Rehabilitation Medicine.

[ref-298498] Hoare Brian J, Wallen Margaret A, Thorley Megan N, Jackman Michelle L, Carey Leeanne M, Imms Christine (2019). Constraint-induced movement therapy in children with unilateral cerebral palsy. Cochrane Database Syst Rev.

[ref-298499] James Sarah, Ziviani Jenny, Ware Robert S, Boyd Roslyn N (2015). Randomized controlled trial of web-based multimodal therapy for unilateral cerebral palsy to improve occupational performance. Developmental Medicine & Child Neurology.

[ref-298500] Kahan Brennan C., Rehal Sunita, Cro Suzie (2015). Risk of selection bias in randomised trials. Trials.

[ref-298501] Portney L.G. (2020). Foundations of Clinical Research: Application to Evidence-Based Practice.

[ref-298502] Gedeborg Rolf, Cline Charles, Zethelius Björn, Salmonson Tomas (2019). Pragmatic clinical trials in the context of regulation of medicines. Upsala Journal of Medical Sciences.

[ref-298503] Garratt A., Schmidt L., Mackintosh A., Fitzpatrick R. (2002). Quality of life measurement: bibliographic study of patient-assessed health outcome measures. BMJ.

[ref-298504] Waters E, Davis E, Boyd R., Reddihough D, Mackinnon A, Graham H (2006). Cerebral Palsy Quality of Life Questionnaire for Children (CPQoL-Child) Manual.

[ref-298505] Newgard Craig D., Lewis Roger J. (2015). Missing data: how to best account for what is not known. JAMA.

[ref-298506] Huque Md Hamidul, Carlin John B., Simpson Julie A., Lee Katherine J. (2018). A comparison of multiple imputation methods for missing data in longitudinal studies. BMC Medical Research Methodology.

[ref-298507] Royston Patrick (2004). Multiple imputation of missing values. The Stata Journal: Promoting communications on statistics and Stata.

[ref-298508] Barnett A. G, Van Der Pols J C, Dobson A J (2005). Regression to the mean: what it is and how to deal with it. International Journal of Epidemiology.

[ref-298509] Power Rosalie, King Catherine, Muhit Mohammad, Heanoy Eamin, Galea Claire, Jones Cheryl, Badawi Nadia, Khandaker Gulam (2018). Health-related quality of life of children and adolescents with cerebral palsy in low- and middle-income countries: a systematic review. Developmental Medicine & Child Neurology.

[ref-298510] Nolte E., Pitchforth E. (2014). What is the evidence on the economic impacts of integrated care?.

[ref-298511] Laragy Carmel, Fisher Karen R., Stancliffe R, Wehmeyer M, Shogren K, Abery B (2020). Choice, Preference, and Disability.

[ref-298512] Gupta Nidhi, Verma Rohan, Dhiman Radha K., Rajsekhar Kavitha, Prinja Shankar (2020). Cost-effectiveness analysis and decision modelling: a tutorial for clinicians. Journal of Clinical and Experimental Hepatology.

